# The association of depressive symptoms and diabetes distress with glycaemic control and diabetes complications over 2 years in newly diagnosed type 2 diabetes: a prospective cohort study

**DOI:** 10.1007/s00125-017-4367-3

**Published:** 2017-08-03

**Authors:** Khalida Ismail, Calum D. Moulton, Kirsty Winkley, John C. Pickup, Stephen M. Thomas, Roy A. Sherwood, Daniel Stahl, Stephanie A. Amiel

**Affiliations:** 1 Department of Psychological Medicine, Institute of Psychiatry, Psychology and Neuroscience, King’s College London, London, SE5 9RJ UK; 2 Division of Diabetes and Nutritional Sciences, Faculty of Life Sciences and Medicine, King’s College London, London, UK; 3 Department of Diabetes and Endocrinology, Guy’s & St Thomas’ NHS Foundation Trust, London, UK; 4 Department of Clinical Biochemistry, King’s College Hospital NHS Foundation Trust, London, UK; 5 Department of Biostatistics, Institute of Psychiatry, Psychology and Neuroscience, King’s College London, London, UK

**Keywords:** Depressive symptoms, Diabetes distress, Glycaemic control, Inflammation, Macrovascular complications, Microvascular complications, Type 2 diabetes

## Abstract

**Aims/hypothesis:**

We examined the associations between depressive symptoms and diabetes distress with glycaemic control and diabetes complications over 2 years, after diagnosis of type 2 diabetes.

**Methods:**

In a multi-ethnic, primary care cohort (*n* = 1735) of adults, all with recent (<6 months) diagnosis of type 2 diabetes, we measured the associations between depressive symptoms (Patient Health Questionnaire-9 [PHQ-9] score ≥10) and diabetes distress (Problem Areas in Diabetes [PAID] score ≥40), with change in 2 year HbA_1c_ as the primary outcome and with incident rates of diabetes complications as secondary outcomes. Multivariate models were used to account for potential confounders.

**Results:**

Of the 1651 participants (95.2%) of the total primary care cohort with available baseline PHQ-9 and PAID scores, mean ± SD age was 56.2 ± 11.1 years, 55.1% were men and 49.1% were of non-white ethnicity; 232 (14.1%) and 111 (6.7%) had depressive symptoms and diabetes distress, respectively. After adjustment for confounders, depressive symptoms were not associated with worsening HbA_1c_. After adjustment for age, sex, ethnicity, vascular risk factors and diabetes treatments, depressive symptoms were associated with increased risk of incident macrovascular complications (OR 2.78 [95% CI 1.19, 6.49], *p* = 0.018) but not microvascular complications. This was attenuated (*p* = 0.09) after adjustment for IL-1 receptor antagonist concentration. Diabetes distress was not associated with worsening HbA_1c_ or incident complications.

**Conclusions/interpretation:**

In the first 2 years of type 2 diabetes, the effect of depressive symptoms and diabetes distress on glycaemic control is minimal. There was, however, an association between depressive symptoms and incidence of macrovascular complications. Elevated innate inflammation may be common to both depression and macrovascular diabetes complications, but these findings require replication.

## Introduction

Depressive symptoms are associated with worse outcomes in the natural history of type 2 diabetes mellitus and are associated with an approximately 60% increased risk of incident disease; conversely, type 2 diabetes increases the risk of incident depressive symptoms by approximately 20% [1, 2]. In established type 2 diabetes, depressive symptoms are associated with a 1.5- to threefold increased risk of diabetes complications and premature mortality [3–5]. The reasons for the adverse effects of depressive symptoms in type 2 diabetes are incompletely understood. A psychological explanation is frequently cited for the relationship, namely that depressive symptoms such as low mood and anhedonia (loss of pleasure in everyday activities) lead to neglect of diabetes self-management and consequent worse glycaemic control [6, 7]. However, pooled data from cross-sectional studies of depressive symptoms and glycaemic control have found only a small effect size (*d* = 0.17) [8]. From the few prospective studies published, the evidence for an association between depressive symptoms and glycaemic control is also weak [9–13]. While treatments for depressive symptoms usually improve depressive symptoms [14, 15], they do not always improve glycaemic control, unless integrated with additional support in diabetes management [16]. This suggests that other mechanisms may also underlie the association between depressive symptoms and type 2 diabetes.

An alternative psychological mechanism is diabetes distress. Diabetes distress is distinguished from depression by referring to the burdens, worries and fears specific to people living with diabetes. Measures of diabetes distress, such as the Problem Areas in Diabetes (PAID) scale [17], show moderate-to-strong correlations with self-report measures of depressive symptoms. However, a large part of the variance in such scales is not explained by depressive symptoms [18]. For example, a longitudinal study of 1567 individuals with diabetes found that 55% of those with diabetes distress did not have likely depression [19], while a factor analysis study reported that depression and distress symptoms could be segregated into two independent factors [20]. Clinically, diabetes distress is significant because it may be a greater psychological barrier to self-management, and therefore to optimising glycaemic control, than depressive symptoms [21, 22] and it is potentially modifiable using psychological therapy [23]. To date, we are aware of only one observational study that has compared depressive symptoms and diabetes distress prospectively in type 2 diabetes, reporting that diabetes distress had a greater effect on worsening glycaemic control than on depressive symptoms [9]. We are not aware of a longitudinal study of the independent effect of diabetes distress on increased risk for diabetes complications.

The prevalence of depressive symptoms and diabetes distress are known to increase with diabetes duration [24, 25], an important confounder that limits the interpretation of previous studies of the effects of depression and diabetes distress on self-management and glycaemic control [11, 26]. Studying these potential effects at the time of diagnosis of type 2 diabetes is a methodologically robust approach, as there is minimal confounding by long duration of type 2 diabetes and it is an important window of opportunity for interventions.

We therefore conducted a multi-ethnic cohort study in primary care to test and compare the associations of depressive symptoms and diabetes distress with change in glycaemic control as the primary outcome, as well as incidence of macro- and microvascular complications as the secondary outcomes, in people with newly diagnosed type 2 diabetes.

## Methods

### Design

The South London Diabetes (SOUL-D) study is a population-based, primary care prospective cohort of people with newly diagnosed type 2 diabetes recruited within 6 months of diagnosis and followed up after 1 and 2 years. Ethical approval was granted by the King’s College Hospital Research Ethics Committee (reference 08/H0808/1) and by Lambeth, Southwark and Lewisham Primary Care Trusts (reference RDLSLB 410). All participants gave written informed consent, including for allowing access to their medical records.

### Setting

The study was set in the inner-city boroughs of Lambeth, Southwark and Lewisham in South London, UK, which collectively have approximately 0.75 million residents from diverse socioeconomic and ethnic backgrounds [27]. All general practitioner (GP) surgeries (*n* = 138) in these boroughs were invited to participate. Local protocols for diagnosis of type 2 diabetes followed WHO criteria [28].

### Study population and case definition

People with a recent (<6 months) diagnosis of type 2 diabetes, aged 18–75 years at diagnosis, were identified from the mandatory electronic diabetes registers of participating surgeries and invited to participate. Exclusion criteria, derived from the medical records, were: dementia, terminal illness, temporary residence and residence outside the catchment area, other types of diabetes and severe end-stage diabetes complications defined as registered blind, receiving dialysis or previous above-knee amputation. Recruitment was conducted between May 2008 and April 2011 and follow-up ended August 2014; further details of the sampling methodology have been described in detail elsewhere [27]. The standardised data collection schedule at baseline consisted of a clinical examination, self-report questionnaires, venepuncture, urine analysis and data extracted from routine medical records, all repeated at 1 and 2 years (±3 months).

### Main outcomes

The primary outcome was HbA_1c_ after 2 years, measured by affinity chromatography (Primus Ultra2; Primus Diagnostics, Kansas City, KS, USA) and reported in both DCCT-derived (%) and IFCC (International Federation of Clinical Chemists)-recommended units (mmol/mol). HbA_1c_ was also measured at 1 year follow-up. If at follow-up there were missing HbA_1c_ data (participant uncontactable or did not want to attend), we used HbA_1c_ from the medical records if it had been collected 3 months before or after the scheduled date. Secondary outcomes were incidence of macrovascular complications (any myocardial infarction [MI], coronary artery bypass graft [CABG], stroke or carotid/limb revascularisation or amputation, derived from the medical records) and microvascular complications (neuropathy, assessed clinically at each study visit by neurothesiometry vibration perception threshold ≥25 V; nephropathy, defined as albumin–creatinine ratio ≥3 mg/mmol, taken from medical records; or retinopathy, assessed using medical record retinal eye screen data, performed annually on all residents with diabetes using digital two-field photography and coded by trained graders as present or absent using the English Retinopathy Minimum Grading System) during follow-up.

### Main explanatory variables

Depressive symptoms were measured by the Patient Health Questionnaire-9 (PHQ-9), a nine-item self-report measure based on the Diagnostic and Statistical Manual-IV diagnostic criteria for major depressive symptoms, with scores ranging from 0 to 27. We used a cut-off score of ≥10 for identifying depressive symptoms, as this has been found to have 88% sensitivity and specificity for major depression compared with clinical interview [29] and was validated in the SOUL-D cohort at baseline [30]. Diabetes distress was measured using the PAID scale [17]. The PAID is comparable across cultures, has high internal and test–retest reliability and is more suitable for both binary and continuous analysis than other measures of diabetes distress [31–33]. We used a score of 40 or higher to define diabetes distress, as this has been found to be around 1 SD above the mean across different studies and has discriminative validity [17, 31]. To account for the possible effect of ‘subthreshold’ symptoms of depression and diabetes distress, we also used both scales as continuous measures.

For participants with <20% missing data for either PHQ-9 or PAID, we used case mean substitution to impute missing values. Briefly, case mean substitution imputes missing values based on the mean scores that are present for that participant [34]. For any participant, this strategy assumes that the score on any missing data point is closely related to the scores on the remaining data points. A major advantage of this technique is that it uses data provided by a case to estimate its own missing data, rather than by using data provided by other cases. Case means substitution has been found to be robust in handling item-level missingness when 20% of data are missing, whether in random or systematic patterns [35].

### Sample size calculation

We estimated that the minimum clinically significant difference in change scores in HbA_1c_ in the depressed group should be 0.5% (5.5 mmol/mol) greater than in the non-depressed group, with a prevalence ratio of depressive symptoms to the control group of 1:8 [3]. Using nQuery Advisor, common SD of 1.77, *α* = 0.05, 90% power and 30% attrition, the total sample required was 1738 people.

### Statistical analyses

Data were analysed using SPSS 22.0 (IBM Corp. Released 2014; IBM SPSS Statistics, Armonk, NY, USA). To assess for potential participation bias, we compared participating and non-participating GP surgeries for list size and index of multiple deprivation (IMD) score, which is an aggregate measure of deprivation across seven domains [36]. The full unit postal code was obtained for each GP surgery and linked with Lower Super Output Area (LSOA), before being assigned an IMD rank. From a total of 32,482 LSOAs in England (UK), a rank of 1 is the most deprived. The baseline characteristics of the sample itself were then stratified by depressive symptoms and diabetes distress status and summarised as mean (SD) or median (interquartile range [IQR]) for normally distributed or skewed data, respectively, or as a count (%) for categorical variables. We calculated the Pearson’s coefficient (*r*) between depressive symptoms and diabetes distress status at baseline.

For the prospective analysis, we first compared the characteristics of participants who had 2 year HbA_1c_ measured with those of participants lost to follow-up. We next compared the proportion of people with depression and diabetes distress at 2 years, stratified by presence or absence of depressive symptoms and diabetes distress at baseline. We then compared 1 and 2 year HbA_1c_ and number of incident complications stratified by presence or absence of depressive symptoms and diabetes distress at baseline. For continuous variables, we used Student’s *t* test for normally distributed data and Mann–Whitney *U* test for skewed data, and for categorical variables we used *χ* ^2^ tests. Our main hypotheses were assessed using multivariable linear regression for HbA_1c_ as outcome and multivariable logistic regression for incident complications as outcome. Finally, we repeated the analyses using continuous PHQ-9 and PAID scores, which were natural log-transformed for multivariate analyses.

#### Covariates

For all multivariate models, we added age, sex and non-white ethnicity as confounders, as these were associated with depression, glycaemic control and complications in the baseline SOUL-D cohort [25], as well as prescription of hypoglycaemic medications and insulin during follow-up. For the outcome 2 year HbA_1c_, we also controlled for baseline HbA_1c_ to measure worsening of glycaemic control over time. For complications, we added the following vascular risk factors to the model: baseline smoking status; baseline systolic blood pressure (mmHg); baseline fasting serum total cholesterol (measured using Siemens Advia 2400 Analyzer, detection limit 0.01 mmol/l); baseline HbA_1c_ and baseline BMI (kg/m^2^). Finally, we added baseline serum concentration of IL-1 receptor antagonist (IL-1RA) to the model as a confounder, as this was the inflammatory cytokine that correlated most strongly with depressive symptoms in a previous study of the cohort [37]. As described in detail previously [37], serum IL-1RA was measured after an overnight fast and analysed using the Randox Evidence Investigator.

## Results

**Fig. 1:**
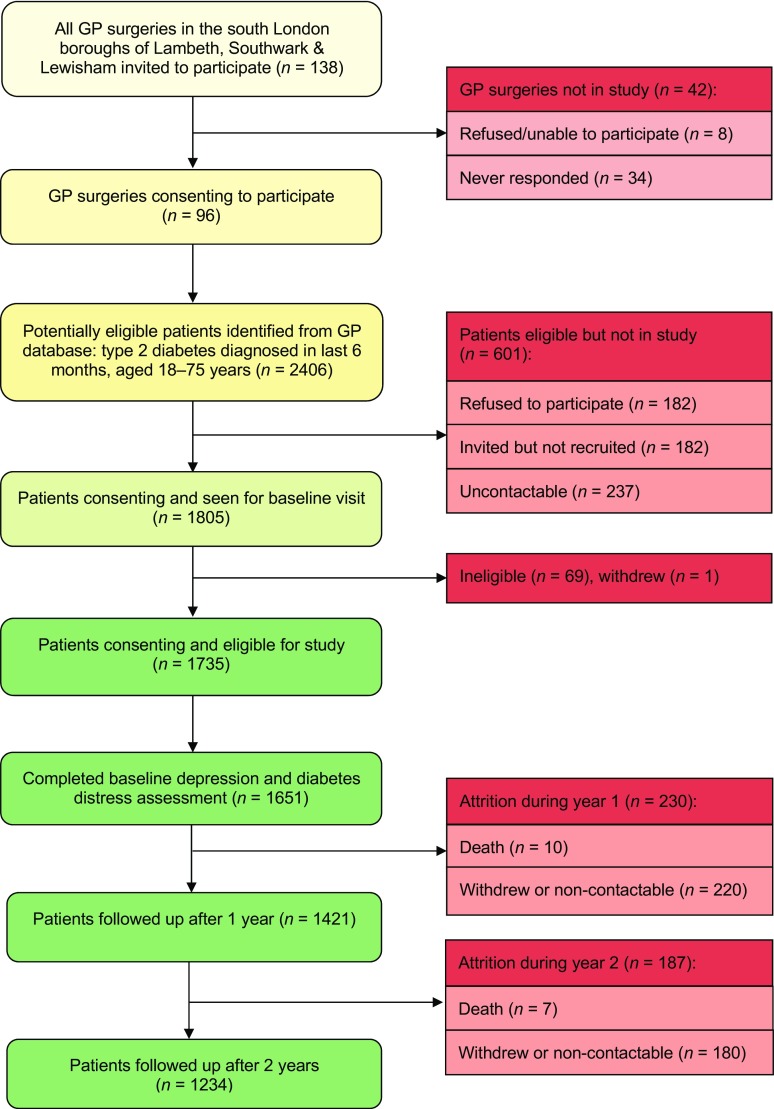
Flow diagram of recruitment and attrition over 2 years in the South London Diabetes Cohort

Ninety-six out of 138 (69.6%) GP surgeries agreed to participate. Participating practices had larger list sizes than those not participating (10,073 ± 4962 vs 5822 ± 3376, *p* < 0.001), but showed no difference in deprivation (IMD rank 7750 ± 4562 vs 8254 ± 4489 *p* = 0.61). From their diabetes registers, a target population of 3008 individuals with newly diagnosed type 2 diabetes were identified during recruitment. Of these, 2406 were potentially eligible and invited to participate and 1735 people consented (Fig. 1).

Of the 1651 people with complete PHQ-9 and PAID assessment at baseline, mean (± SD) age was 56.2 ± 11.1 years, 55.1% were men, 49.1% were of non-white ethnicity and mean HbA_1c_ was 6.99 ± 1.42% (52.9 ± 10.7 mmol/mol). Of these, 232 (14.1%) and 111 (6.7%) participants had depressive symptoms and diabetes distress, respectively, at baseline. Of those with diabetes distress at baseline, 59 (53.2%) had comorbid depressive symptoms, while 59 (25.4%) of those with depressive symptoms had comorbid diabetes distress. The resulting correlation between diabetes distress status and depressive symptoms status was *r* = 0.30 (*p* < 0.001).

### Baseline comparisons

At baseline, participants with depressive symptoms were younger, more likely to be women, had higher BMI, were more likely to smoke and had higher baseline IL-1RA concentration. Participants with diabetes distress were younger, more likely to be women and of non-white ethnicity and had lower systolic BP and higher IL-1RA concentration. There were no significant differences in glycaemic control, total cholesterol, diastolic BP or prescription of oral hypoglycaemic medication when participants were stratified by depressive symptoms or diabetes distress (Table 1).

**Table 1:** Baseline characteristics of the SOUL-D cohort

Characteristic	Total (*n*)^a^	Depressive symptoms (*n* = 232)	No depressive symptoms (*n* = 1419)	*p* value^b^	Diabetes distress (*n* = 111)	No diabetes distress (*n* = 1540)	*p* value^b^
Age (years), mean ± SD	1651	53.07 ± 10.47	56.72 ± 11.07	<0.001	49.82 ± 10.70	56.67 ± 10.95	<0.001
Sex, *n* (%)							
Male	909	111 (47.84)	798 (56.24)	0.018	42 (37.84)	867 (56.30)	<0.001
Female	742	121 (52.16)	621 (43.76)		69 (62.16)	673 (43.70)	
Ethnicity, *n* (%)							
White	840	116 (50.00)	724 (51.02)	0.77	36 (32.43)	804 (52.21)	<0.001^c^
Black	643	82 (35.34)	561 (39.53)		52 (46.85)	591 (38.38)	
South Asian/other	168	34 (14.66)	134 (9.44)		23 (20.72)	145 (9.42)	
Smoker status, *n* (%)							
Smoker	327	72 (31.86)	255 (18.44)	<0.001	25 (23.58)	302 (20.09)	0.40
Non-smoker	1282	154 (68.14)	1128 (81.56)		81 (76.42)	1201 (79.91)	
BMI (kg/m^2^), mean ± SD	1648	33.34 ± 7.29	31.78 ± 6.36	0.002	32.83 ± 6.08	31.94 ± 6.55	0.14
Systolic BP (mmHg), mean ± SD	1588	133.67 ± 18.88	136.31 ± 17.29	0.055	132.70 ± 16.68	136.19 ± 17.58	0.042
Diastolic BP (mmHg), mean ± SD	1588	83.82 ± 10.98	82.81 ± 10.78	0.21	83.70 ± 9.00	82.89 ± 10.92	0.38
HbA1c (%), mean ± SD	1551	7.11 ± 1.37	6.97 ± 1.42	0.17	7.20 ± 1.61	6.98 ± 1.40	0.17
HbA_1c_ (mmol/mol), mean ± SD	1551	54.2 ± 10.44	52.70 ± 10.74	0.17	55.20 ± 12.34	52.80 ± 10.59	0.17
Mean total cholesterol (mmol/l), mean ± SD	1518	4.68 ± 1.21	4.57 ± 1.06	0.22	4.59 ± 1.01	4.58 ± 1.09	0.92
IL-1RA (ng/l), median (IQR)	1190	498.10 (337.30–785.30)	428.70 (282.40–660.00)	0.002	506.55 (340.48–772.60)	433.20 (288.65–684.05)	0.047
Prescribed any oral hypoglycaemic medication, *n* (%)							
Yes	879	136 (59.65)	743 (53.03)	0.089	71 (64.55)	808 (53.19)	0.022
No	750	92 (40.35)	658 (46.97)		39 (35.45)	711 (46.81)	
Depressive symptoms: PHQ-9 ≥10, *n* (%)							
Yes	232	–	–		59 (53.15)	173 (11.23)	<0.001
No	1419	–	–		52 (46.85)	1367 (88.77)	
Diabetes distress: PAID ≥40, *n* (%)							
Yes	111	59 (25.43)	52 (3.66)	<0.001	–	–	
No	1540	173 (74.57)	1367 (96.34)		–	–	

The cohort was stratified by: (1) presence/absence of depressive symptoms at baseline as measured by the PHQ-9 and (2) presence/absence of diabetes distress at baseline as measured by the PAID scale

^a^There are missing data for some variables resulting in different percentages; the total number of individuals for each variable is therefore given

^b^Parametric continuous data were compared using Student’s *t* test; non-parametric continuous data were compared using Mann–Whitney *U* test; categorical variables were compared using *χ* ^2^ tests

^c^Compared with non-white ethnicity

### Comparison of dropouts with non-dropouts at 2 years

At 2 years, those who had HbA_1c_ measured (*n* = 1234) vs those who did not (*n* = 417) were older (56.8 ± 10.9 years vs 54.5 ± 11.5 years, *p* < 0.001), had lower baseline HbA_1c_ (6.91 ± 1.36% [52.0 ± 10.2 mmol/mol]) vs 7.23 ± 1.56% [55.5 ± 12.0 mmol/mol], *p* < 0.001) and were more often of white ethnicity (53.6% vs 42.9%, *p* < 0.001). There were no differences in sex (*p* = 0.52), baseline depressive symptoms status (*p* = 0.064), diabetes distress status (*p* = 0.37), smoking status (*p* = 0.92), prescription of hypoglycaemic medication (*p* = 0.19), serum cholesterol (*p* = 0.67), BMI (*p* = 0.94), systolic BP (*p* = 0.96) or diastolic BP (*p* = 0.71) (data not shown).

### Course of depressive symptoms and diabetes distress

Fifty-four per cent of people with depressive symptoms at baseline still had depressive symptoms 2 years later, while the 2 year prevalence of depressive symptoms (13.8%) was similar to that seen at baseline. Conversely, only 17.1% of people with diabetes distress at baseline still had diabetes distress 2 years later, while the overall prevalence of diabetes distress fell to 2.4% after 2 years.

### HbA_1c_

In univariate analysis, depressive symptoms at baseline were not associated with elevated HbA_1c_ after 1 year. Conversely, depressive symptoms at baseline were associated with elevated HbA_1c_ after 2 years (Table 2) but this was attenuated after adjustment for baseline HbA_1c_, age, sex, ethnicity and diabetes treatments (Table 3). Baseline diabetes distress was not associated with elevated 1 year or 2 year HbA_1c_ in univariate or adjusted models (Table 2 and Table 3). Continuous depressive symptoms and diabetes distress were both associated with elevated 2 year HbA_1c_ in univariate analysis. Again, however, differences were attenuated after adjusting for confounders (Table 3).

**Table 2:** Outcomes for the SOUL-D cohort

Outcome	Total (*n*)^a^	Depressive symptoms (*n* = 232)	No depressive symptoms (*n* = 1419)	*p* value^b^	Diabetes distress (*n* = 111)	No diabetes distress (*n* = 1540)	*p* value^b^
Psychological							
Depressive symptoms: PHQ-9 ≥10 at 2 years, *n* (%)							
Yes	162	82 (54.30)	80 (7.82)	<0.001	34 (45.95)	128 (11.63)	<0.001
No	1012	69 (45.70)	943 (92.18)		40 (54.05)	972 (88.37)	
Diabetes distress: PAID ≥40 at 2 years, *n* (%)							
Yes	28	17 (11.64)	11 (1.09)	<0.001	12 (17.14)	16 (1.47)	<0.001
No	1128	129 (88.36)	999 (98.91)		58 (82.86)	1070 (98.53)	
Glycaemic control							
1 year HbA1c (%), mean ± SD	1421	6.98 (1.30)	6.89 (1.27)	0.38	6.79 (1.34)	6.91 (1.27)	0.69
1 year HbA_1c_ (mmol/mol), mean ± SD	1421	52.80 (9.83)	51.80 (9.55)	0.38	51.0 (10.3)	51 (9.56)	0.69
2 year HbA1c (%), mean ± SD	1234	7.13 (1.32)	6.89 (1.28)	0.035	7.14 (1.43)	6.91 (1.28)	0.16
2 year HbA_1c_ (mmol/mol), mean ± SD	1234	54.40 (10.07)	51.80 (9.62)	0.035	54.50 (10.87)	52.00 (9.63)	0.16
Complications							
Incident MI or CABG by 2 years, *n* (%)							
Yes	24	7 (4.12)	17 (1.58)	0.031	2 (2.50)	22 (1.89)	0.40
No	1221	163 (95.88)	1058 (98.42)		78 (97.50)	1143 (98.91)	
Incident stroke by 2 years, *n* (%)							
Yes	12	3 (1.78)	9 (0.84)	0.26	0 (0.00)	12 (1.03)	1.0
No	1230	166 (98.22)	1064 (99.16)		80 (100.00)	1150 (98.97)	
Any incident carotid/limb revascularisation or amputation by 2 years, *n* (%)							
Yes	13	2 (1.18)	11 (1.02)	0.86	1 (1.25)	12 (1.03)	0.79
No	1231	168 (98.82)	1063 (98.98)		79 (98.75)	1152 (98.97)	
Any incident macrovascular complication by 2 years, *n* (%)							
At least one	40	10 (5.88)	30 (2.79)	0.037	3 (3.75)	37 (3.17)	0.50
None	1207	160 (94.12)	1047 (97.21)		77 (96.25)	1130 (96.83)	
Retinopathy present by 2 years, *n* (%)							
Yes	162	27 (16.67)	135 (13.26)	0.24	15 (17.65)	147 (13.42)	0.39
No	1018	135 (83.33)	883 (86.74)		70 (82.35)	948 (86.58)	
Neuropathy present by 2 years, *n* (%)							
Yes	113	13 (9.70)	100 (10.49)	0.78	3 (4.23)	110 (10.83)	0.13
No	974	121 (90.30)	853 (89.51)		68 (95.77)	906 (89.17)	
Nephropathy present by 2 years (%)							
Yes	156	22 (16.06)	134 (15.40)	0.90	12 (17.65)	144 (15.33)	0.48
No	851	115 (83.94)	736 (84.60)		56 (82.35)	795 (84.67)	
Any microvascular complication by 2 years, *n* (%)							
At least one	388	55 (30.73)	333 (28.83)	0.60	29 (31.52)	359 (28.90)	0.54
None	946	124 (69.27)	822 (71.17)		63 (68.48)	883 (71.10)	
Diabetes treatment							
Prescribed insulin at 2 years (%)							
Yes	54	14 (7.65)	40 (3.43)	0.008	7 (7.78)	47 (3.73)	0.065
No	1296	169 (92.35)	1127 (96.57)		83 (92.22)	1213 (96.27)	
Prescribed diabetes medication at 2 years, *n* (%)							
Yes	894	137 (74.86)	757 (64.87)	0.008	73 (80.22)	821 (65.21)	0.004
No	456	46 (25.14)	410 (35.13)		18 (19.78)	438 (34.79)	

The cohort was stratified by: (1) presence/absence of depressive symptoms at baseline as measured by the PHQ-9 and (2) presence/absence of diabetes distress status at baseline as measured by the PAID scale

^a^There are missing data for some variables resulting in different percentages; the total number of individuals for each variable is therefore given

^b^Parametric continuous data were compared using Student’s *t* test; non-parametric continuous data were compared using Mann–Whitney *U* test; categorical variables were compared using χ^2^ tests

**Table 3:** Unadjusted and adjusted associations of depressive symptoms and diabetes distress at baseline with 2 year biomedical outcomes

Baseline predictor	2 year HbA_1c_, %		Any incident macrovascular complication (%)			Any incident microvascular complication (%)		
	Unadjusted	Adjusted^a^	Unadjusted	Partially adjusted^b^	Fully adjusted^c^	Unadjusted	Partially adjusted^b^	Fully adjusted^c^
Depressive symptoms: PHQ-9 ≥10								
β (95% CI) or OR (95% CI)^d,e^	β 0.23 (0.02, 0.45)	β 0.068 (−0.13, 0.26)	OR 2.18 (1.05, 4.55)	OR 2.78 (1.19, 6.49)	OR 2.29 (0.88, 5.95)	OR 1.10 (0.78, 1.54)	OR 1.33 (0.90, 1.96)	OR 1.32 (0.85, 2.05)
*p* value	0.031	0.49	0.037	0.018	0.088	0.60	0.15	0.22
Diabetes distress: PAID ≥40								
β (95% CI) or OR (95% CI)^d,e^	β 0.23 (0.06, 0.53)	β 0.032 (−0.24, 0.30)	OR 1.19 (0.36, 3.95)	OR 2.66 (0.73, 9.69)	OR 1.09 (0.14, 8.82)	OR 1.13 (0.72, 1.79)	OR 1.28 (0.76, 2.16)	OR 1.45 (0.79, 2.67)
*p* value	0.12	0.82	0.78	0.14	0.94	0.59	0.36	0.23
Total PHQ-9 score								
β (95% CI) or OR (95% CI)^d,e^	β 0.012 (0.05, 0.20)	β 0.02 (−0.05, 0.09)	OR 1.39 (1.00, 1.94)	OR 1.62 (1.12, 2.35)	OR 1.49 (0.99, 2.21)	OR 1.03 (0.91, 1.16)	OR 1.08 (0.93, 1.24)	OR 1.08 (0.92, 1.27)
*p* value	0.001	0.63	0.05	0.010	0.051	0.64	0.31	0.34
Total PAID score								
β (95% CI) or OR (95% CI)	β 0.11 (0.06, 0.17)	β 0.02 (−0.04, 0.08)	OR 0.98 (0.75, 1.26)	OR 1.12 (0.83, 1.51)	OR 0.97 (0.70, 1.34)	OR 1.04 (0.94, 1.14)	OR 1.09 (0.97, 1.22)	OR 1.08 (0.95, 1.23)
*p* value	<0.001	0.51	0.85	0.47	0.87	0.45	0.14	0.24
Total number in model	1234	1147	1247	1049	848	1334	1118	884

^a^Multivariable linear regression model (outcome: 2 year HbA_1c_) adjusted for baseline HbA_1c_, age, sex, non-white ethnicity, prescription of oral hypoglycaemic medication at 2 years and prescription of insulin at 2 years

^b^Multivariable logistic regression model (outcome: any incident complication during the 2 year follow-up) adjusted for age, sex, non-white ethnicity, baseline BMI, baseline systolic BP, baseline smoking status, baseline serum cholesterol, baseline HbA_1c_, prescription of oral hypoglycaemic medication at 2 years and prescription of insulin at 2 years

^c^Multivariable logistic regression model (outcome: any incident complication during the 2 year follow-up) adjusted for age, sex, non-white ethnicity, baseline BMI, baseline systolic BP, baseline smoking status, baseline serum cholesterol, baseline HbA_1c,_ prescription of oral hypoglycaemic medication at 2 years, prescription of insulin at 2 years and baseline IL-1RA concentration (natural log-transformed)

### Complications

In univariate analysis, there was a positive association between baseline depressive symptoms and incident macrovascular complications. This was largely explained by incident MI, although numbers were low (Table 2). Notably, this association remained significant (OR 2.78) even after adjustment for age, sex, ethnicity, vascular risk factors and diabetes treatments, but was attenuated after adjustment for baseline IL-1RA concentration (Table 3). Similar findings were observed for continuous PHQ-9 score. For diabetes distress, there was no association with incident macrovascular complications, whether measured categorically or continuously (Table 2 and Table 3). In univariate or adjusted models, there was no association between either depressive symptoms or diabetes distress (measured categorically or continuously) and incident microvascular complications (Table 2 and Table 3).

## Discussion

The SOUL-D cohort is a multi-ethnic, population-based primary care cohort of people with newly diagnosed type 2 diabetes, designed and powered to test the effects of depressive symptoms on proxy markers of cardiovascular risk. Unexpectedly, we found that neither depressive symptoms nor diabetes distress were associated with worsening HbA_1c_ after 2 years. Despite a low number of overall events, depressive symptoms at diagnosis of type 2 diabetes were associated with greater risk for macrovascular complications during 2 year follow-up. This occurred independently of age, sex, ethnicity and vascular risk factors but was attenuated after adjustment for serum IL-1RA. Because all the participants were recruited within 6 months of diagnosis, confounding by duration of diagnosed diabetes was minimised.

### Comparison with other studies

#### Prevalence of diabetes distress and depressive symptoms

The low prevalence of diabetes distress in the SOUL-D cohort is comparable with other primary care samples [38]. Hospital cohorts have generally reported much higher prevalence of diabetes distress [22, 38] but this may relate to a bias towards people with more complex and complicated diabetes in these services. Like other studies, we found that depressive symptoms and diabetes distress, although correlated, overlapped in only a minority of individuals [19].

#### Glycaemic control

After adjustment for confounders including baseline glycaemic control, neither depressive symptoms nor diabetes distress were associated with worse glycaemic control after 2 years in our study. In two previous prospective studies of depressive symptoms, but not diabetes distress, any differences in glycaemic control were likewise negated by adjusting for baseline glycaemic control [10, 11]. In comparing diabetes distress and depressive symptoms, a study of 3305 Japanese individuals with type 2 diabetes reported that diabetes distress was associated with greater odds of poor glycaemic control (HbA_1c_ ≥8%) than depressive symptoms [26]. However, that cohort differed from ours by the long-established diabetes of its participants (mean 13.8 years, 41% receiving insulin therapy). Likewise, a study of 627 individuals in a secondary care setting in the Netherlands reported that diabetes distress was more strongly associated with glycaemic control than depressive symptoms [22]. Both studies were limited by their cross-sectional design. Only one previous observational study in a type 2 diabetes cohort has compared depressive symptoms and diabetes distress for their prospective associations with glycaemic control [9]. In this study, Fisher and colleagues reported a significant association between diabetes distress and worsening glycaemic control, an association not demonstrated for depressive symptoms. Although comparable with ours in recruiting a multi-ethnic, primary care cohort, the Fisher study was limited by a smaller sample size (*n* = 506), shorter follow-up, longer mean duration of diabetes (8.1 years) and lack of data on incident complications.

#### Complications

A previous meta-analysis found that depressive symptoms were associated with both macrovascular and microvascular complications in individuals with type 1 and type 2 diabetes [4]. Several studies have demonstrated that depressive symptoms are associated with incident stroke in individuals with type 2 diabetes [39, 40]. For diabetes distress, one previous prospective cohort study reported that general distress (rather than diabetes-specific distress) was associated with a 1.7-fold increased risk of cardiovascular disease over time [41]. To date, however, we are not aware of any study that has investigated the prospective association between diabetes distress and depressive symptoms with incident complications.

### Interpretation

Our findings were unexpected. If the effect of depression resulted from a behavioural mechanism such as self-neglect and poor adherence to medication, it is reasonable that the consequences would manifest as poorer glycaemic control within the first 2 years of diagnosis. We did not observe this but we did observe an effect of depression on macrovascular disease. While this may reflect pre-existing vulnerability to cardiovascular disease, it could be that depression is also a proxy marker for future cardiovascular risk. Although the number of events was low, we found that the association between depressive symptoms and incident macrovascular disease occurred independently of other known vascular risk factors and glycaemic control. When we further controlled for baseline IL-1RA concentration, the association between depressive symptoms and macrovascular disease was attenuated, pointing towards a possible inflammatory mechanism. There is compelling evidence that elevated inflammation is on the causal pathway for the pathogenesis of type 2 diabetes and subsequent macrovascular complications [42, 43]. Intervention studies that would allow testing of the directionality of the association between depressive symptoms, inflammation and biomedical outcomes are needed. Future studies should take into account wider contextual factors, such as frequency of contact with healthcare professionals, access to diabetes education and access to mental health services.

### Limitations

Our study has several limitations. Despite its inner-city setting with high overall levels of deprivation, we were able to achieve nearly 70% GP participation. Although there is a risk of selection bias compared with those not participating, there was no difference in deprivation indicators between those surgeries participating and those not. There is a risk of attrition bias due to dropout at 2 years, although neither of the explanatory variables were significant predictors of attrition. The low prevalence of diabetes distress may have restricted power when testing its associations. We used a self-report measure of depressive symptoms, which is well suited to epidemiological studies but will likely result in more cases of depressive symptoms when compared with a diagnostic interview. Although our findings were supported by both categorical and continuous measures of depressive symptoms, the overall number of macrovascular events during follow-up was low. We did not control for previous cardiovascular disease as this variable may be collinear, being on the causal pathway to future cardiovascular disease, and addition of this covariate would have reduced the number of events per cell. Likewise, because of low numbers of events, effect modification analysis stratified by inflammation status was not possible and IL-1RA could only be included as a confounder in the analyses. The latter two limitations will only be overcome with longer-term follow-up, by which time a greater number of macrovascular events is expected.

### Conclusion

We conclude from our study that neither depressive symptoms nor diabetes distress is associated with worsening glycaemic control over 2 years. Conversely, depressive symptoms are independently associated with increased risk of incident macrovascular disease in the first 2 years of type 2 diabetes. As the overall number of events was low, longer-term prospective studies and intervention studies are now needed to test whether depressive symptoms at diagnosis of type 2 diabetes are a modifiable target.

## Abbreviations

CABG: Coronary artery bypass graft
GP: General practitioner
IL-1RA: IL-1 receptor antagonist
IMD: Index of multiple deprivation
IQR: Interquartile range
LSOA: Lower Super Output Area
MI: Myocardial infarction
PAID: Problem Areas in Diabetes
PHQ-9: Patient Health Questionnaire-9
SOUL-D: South London Diabetes
